# Efficacy of Endopyelotomy for Secondary Pelvi-Ureteric Junction (PUJ) Obstruction in Terms of Improvement in Renal Function

**DOI:** 10.7759/cureus.54728

**Published:** 2024-02-22

**Authors:** Rizwan Ullah, Wasim Ali, Malik Adil Mehmood, Wajid Ali, Muhammad Bilal Hameed, Mohib Ullah, Abdullah Khan Zada, Khalid Naveed Khan

**Affiliations:** 1 Urology, Institute of Kidney Diseases, Peshawar, PAK; 2 Internal Medicine, Swat Medical Complex, Saidu Sharif, PAK; 3 Surgery, Hayatabad Medical Complex Peshawar, Peshawar, PAK; 4 Urology, Pakistan Institute of Medical Sciences, Islamabad, PAK; 5 Cardiology, Hayatabad Medical Complex Peshawar, Peshawar, PAK; 6 Surgery, Lady Reading Hospital, Peshawar, PAK

**Keywords:** renal functions, dtpa renal scintigraphy, ureteropelvic junction obstruction, secondary pelvi-ureteric junction obstruction, endopyelotomy

## Abstract

Objective

The objective of this study is to measure renal function improvement after endopyelotomy for secondary pelvi-ureteric junction (PUJ) obstruction using technetium-99m diethylene-triamine-pentaacetate (DTPA) renal scintigraphy.

Material and methods

This descriptive study was carried out at the Department of Urology, Institute of Kidney Diseases, Peshawar, Pakistan from June 1, 2021, to May 31, 2023. The study included 118 secondary PUJ blockage patients who underwent endopyelotomy. Patient demographics, clinical history, and preoperative imaging findings were obtained. DTPA renal scintigraphy assessed renal function improvement postoperatively at intervals to determine the efficacy of endopyelotomy.

Results

The majority of the patients included in the study were male (n=65, 55.1%). The average age of the patients was 45.2 years, with the majority falling within the age range of 46-60 years (n=42, 35.6%). All patients had ultrasonography and computed tomography imaging done, and preoperative renal functions were obtained. Comorbidities included hypertension in 32 (27.12%) and diabetes in 18 (15.25%). DTPA renal scintigraphy showed improved renal function in 81.35% of patients at three months, 88.13% at six months, and 83.05% at 12 months; 15.3% of patients needed further treatments, and 5.1% had PUJ blockage recurrence.

Conclusion

This study offers significant insights into the results and complexities of endopyelotomy in patients suffering from PUJ blockage. The findings indicate that the technique efficiently enhances kidney function and alleviates symptoms in most patients. However, the study also emphasizes the need to monitor patients undergoing this procedure.

## Introduction

Secondary pelvi-ureteric junction (PUJ) blockage is a complicated and intricate problem in urology. It often necessitates surgical treatment to restore normal urine flow [1]. Endopyelotomy is a minimally invasive surgery as a possible treatment for secondary PUJ obstruction [2,3]. In the past, many surgical methods, including open pyeloplasty and laparoscopic and endoscopic procedures, have been used to treat this condition [4]. Endopyelotomy has received attention as a feasible option for some circumstances due to its benefits in terms of less invasiveness and faster recovery [5,6]. The causes of secondary PUJ blockage are numerous, such as scarring after surgery, stone disease, and external compression [7].

Comprehending the fundamental reasons is essential for customizing suitable therapies and maximizing patient outcomes [8]. The main objective is to relieve blockage, although the maintenance and enhancement of renal function are of utmost importance in the overall therapeutic approach. In a study conducted to assess renal function improvement after antegrade endopyelotomy, improvement was noted in 14 cases, no change in 22, and deterioration in two out of a total of 38 cases [9,10].

This study intends to add to the developing field of urological therapies by evaluating the functional effects of endopyelotomy for secondary PUJ blockage. The assessment of functional improvement was conducted using technetium-99m diethylene triamine pentaacetate (DTPA) renal scintigraphy, a dependable method for evaluating renal function and drainage.

This study examines the effects of endopyelotomy on renal function in 118 secondary PUJ blockage patients to determine its effectiveness and feasibility. The results may help adjust secondary PUJ blockage treatment algorithms by revealing when endopyelotomy is most suitable. Secondary PUJ blockage treatment should balance intervention invasiveness with therapeutic effectiveness to improve patient outcomes and quality of life.

## Materials and methods

This descriptive study was conducted at the Department of Urology, Institute of Kidney Diseases, Peshawar, Pakistan, between June 1, 2021, and May 31, 2023, after approval from the Institutional Research and Ethical Board of Hayatabad Medical Complex Peshawar (approval number: 353).

Inclusion criteria were patients in the age range of 18-75 years with confirmed secondary PUJ blockage based on imaging and patients who were willing to undergo endopyelotomy. Patients with contraindications to endoscopic procedures or those who had hypersensitivity to DTPA, patients with a prior history of other interventions for secondary PUJ blockage, and patients with chronic renal failure were excluded. A total of 118 patients were included in the study on the basis of these criteria.

Every patient underwent a thorough pre-operative evaluation, which included an extensive medical history review, a comprehensive physical examination, renal function tests, imaging studies (ultrasound, computed tomography), and technetium-99m DTPA renal scintigraphy to establish the initial renal function. Procedure details, such as the length of the surgery, any complications that occurred during the operation, and the need for further treatments, were documented. Endopyelotomy was performed using the standard antegrade percutaneous approach to get access to the obstructed segment of PUJ over a guidewire placed after obtaining a pyelogram. The obstructed segment was serially dilated with dilating catheters to 14 Fr followed by stent placement.

Patients were monitored periodically after surgery, and DTPA renal scintigraphy was conducted at specific time points (three months, six months, and 12 months) to evaluate changes in renal function. Follow-up renal functions were compared with baseline pre-operative measurements. Less than 50% increase in renal functions was called no improvement, and more than 50% increase in renal functions was called improvement, which was the study's primary outcome. Secondary outcomes included the complications that were experienced during the procedure, such as bleeding, and the need for ancillary interventions such as additional port site and post-post-procedure infections.

Data was analyzed using IBM SPSS Statistics for Windows, Version 24.0 (Released 2016; IBM Corp., Armonk, New York, United States). Continuous data was presented as mean±SD or median (interquartile range (IQR)). Categorical data was presented as frequencies and percentages. Continuous variables were compared using the student or Man-Whitney U test depending upon the normality of the data assessed using the Shapiro-Wilk test. Categorical variables were compared using the Chi-square test or Fisher exact test. Patient demographics and baseline characteristics were summarized using descriptive statistics. P-value ≤ 0.05 was considered statistically significant.

## Results

The study sample included a slightly more greater proportion of males (n=65, 55.1%), than females (n=53, 44.9%). The average age of the patients was 45.2 years, with the majority falling within the age range of 46-60 years (n=42, 35.6%), as shown in Table 1.

**Table 1:** Demographic Characteristics of Study Population (N = 118)

Demographic Parameters	Number of Patients (n)	Percentages (%)
Gender		
Male	65	55.1%
Female	53	44.9%
Age (mean ± S.D)	45.2 ± 6.8 years	
Age Groups (years)		
18-30	12	10.2%
31-45	28	23.7%
46-60	42	35.6%
61-75	30	25.4%
76 and above	6	5.1%

Preoperative characteristics and renal functions of the study cohort were documented. Diagnostic modalities included ultrasonography in 118 (100%) and computed tomography of kidneys, ureters, and bladder (CT KUB) in 92 patients (77.9%). The mean preoperative differential renal function on DTPA scan was 35.4 ± 8.7%. Other results included hypertension in 32 (27.12%) and diabetes in 18 (15.25%) patients. Secondary PUJ blockage was most often caused by surgical scarring (n=42, 35.59%), stone disease (n=52, 44.07%), and extrinsic compression (n=24, 20.34%). Table 2 illustrates information about patients' preoperative state and PUJ blockage causes.

**Table 2:** Preoperative Characteristics and Baseline Renal Functions (N =118) PUJ: pelvi-ureteric junction

Parameters	Number of Patients (n)	Percentages (%)
Preoperative differential renal function (Mean±S.D)	35.4 ± 8.7 ml/min	
Diagnostic imaging findings		
Ultrasound	118	100.0%
CT scan	92	77.97%
Other relevant findings (comorbidities)		
Hypertension	32	27.12%
Diabetes	18	15.25%
Etiology of secondary PUJ obstruction		
Postoperative scarring	42	35.59%
Stone disease	52	44.07%
Extrinsic compression	24	20.34%

The average time of the endopyelotomy surgery was 118 minutes. Intraoperative complications occurred in 15 (12.7%) patients, with eight patients (6.8%) needing further auxiliary interventions. These facts provide light on the duration and complexity of the endopyelotomy process, as well as the possible dangers and extra interventions required during the surgery (Table 3).

**Table 3:** Intraoperative Details and Procedural Characteristics (N = 118)

Parameters	Number of Patients (n)	Percentages (%)
Duration of endopyelotomy (Mean±S.D) 118±9.13 minutes		
Intraoperative complications	15	12.7%
Need for ancillary procedures	8	6.8%

Table 4 presents postoperative follow-ups that occurred three, six, and 12 months following endopyelotomy. DTPA renal scintigraphy showed renal function improvement in 81.35% of patients at three months, 88.13% at six months, and 83.05% at 12 months. Few individuals (5.93-18.64%) had stable renal function at each follow-up. Additionally, 75.42% of patients reported clinical symptom eradication and 17.79% improvement. These data show that endopyelotomy improves kidney function and relieves symptoms in most individuals.

**Table 4:** Postoperative Follow-up and Changes in Renal Functions of Patients Who Underwent Endopyelotomy (primary outcomes) (N = 118) DTPA: Diethylene-triamine-phenacetate

Follow-up Interval and Renal Function Status	Number of Patients (n)	Percentages (%)
3-month DTPA renal scintigraphy		
Improved	96	81.35%
Stable	22	18.65%
6-month DTPA renal scintigraphy		
Improved	104	88.13%
Stable	14	11.87%
12-month DTPA renal scintigraphy		
Improved	98	83.05%
Stable	20	5.93%
Resolution of clinical symptoms		
Resolved	89	75.4%
Improved	21	17.8%
Stable/Increased	08	6.8%

The study population also had secondary outcomes and problems evaluated; 15.3% of patients needed further treatments, 5.1% had PUJ blockage recurrence, and 10.2% had other complications. These findings indicate that endopyelotomy patients may face hazards and need continuing treatment (Table 5).

**Table 5:** Intraoperative and Post-Operative Complications of Patients Who Underwent Endopyelotomy (Secondary Outcomes and Complications) PUJ: Pelvi-ureteric junction

Secondary Outcomes/Complications	Number of Patients (n)	Percentages (%)
Need for additional Interventions	18	15.3%
Recurrence of PUJ obstruction	6	5.1%
Complications (infections)	12	10.2%

## Discussion

The research findings unequivocally establish the efficacy of endopyelotomy in enhancing renal function and alleviating symptoms in patients suffering from PUJ blockage. Most patients had improved renal function at every subsequent assessment, whereas a few demonstrated consistent function. These findings align with other research that has shown comparable rates of renal function improvement after endopyelotomy [11]. The findings of this study also align with prior research, indicating that 70-90% of patients had the resolution or improvement of symptoms, thereby providing more evidence of symptom alleviation [12,13]. This underscores the efficacy of endopyelotomy in enhancing renal function and alleviating symptoms in patients with PUJ blockage.

However, the rate of progress seen in this investigation was slightly lower in comparison to other studies, which documented rates ranging from 90% to 95% [14]. This phenomenon may be attributed to the incorporation of patients with intricate situations, such as those with postoperative scarring, which might potentially exhibit a lower rate of success when compared to other causes.

The incidence of complications such as infections in our study sample was 10.2%, which is somewhat more significant in comparison to earlier investigations, which have shown rates ranging from 5% to 8%[15]. The possible reason for this might be the incorporation of patients with more intricate conditions, together with the extended duration of observation in this research (12 months as opposed to six months in previous studies). However, most of the issues were minor and did not need further therapies, aligning with earlier research findings.

The need for supplementary therapies in 15.3% of patients is greater in comparison to other investigations, which have shown rates ranging from 10% to 12% [16]. However, the risk of recurrence (5.1%) aligns with earlier studies that have demonstrated rates ranging from 3% to 6% [15]. This underscores the need for continuous supervision and subsequent monitoring in patients who undergo endopyelotomy.

Overall, this study's findings unequivocally establish the importance of endopyelotomy in the management of secondary PUJ obstruction with a better safety profile. However, there are certain limitations of the study, like a small sample size, which may limit the results' applicability. Similarly, only patients from one facility were included. Therefore, the findings may not apply elsewhere. Finally, endopyelotomy was not compared to open or laparoscopic PUJ obstruction surgery. This makes comparing endopyelotomy effectiveness and safety to other therapies challenging. Future studies may include a comparison group to evaluate PUJ-blocking treatment.

## Conclusions

This research offers significant insights into the effectiveness and complexities of endopyelotomy in patients suffering from PUJ blockage. The findings indicate that this technique is very efficient in enhancing kidney function and alleviating symptoms in most patients. However, the research also emphasizes the possible risk of complications and the need for continuous supervision in patients who undergo endopyelotomy. Additional research with large sample sizes and extended follow-up periods might provide more detailed data about the results and possible issues associated with this technique.
